# Innovations and African Health Sciences: telling it beyond our environs

**DOI:** 10.4314/ahs.v25i2.1

**Published:** 2025-06

**Authors:** James K Tumwine

We bring you this June 2025 issue of *African Health Sciences* with a key theme of innovation. Hence Ugandan scientists describe their work on tuberculosis and human hair^1^ followed by unique work on onchocerciasis^2^ in Ethiopia and antimicrobial resistance in Ghana^3^. Other papers are on Hepatitis B in Tanzania 4, and use of lactobacilli in treatment of virus enteritis^5^. These are followed by innovation in the diagnosis of micrognathia using real time doppler ultra sound 6 and use of laser for treatment of cervical intra epithelial high risk human papilloma virus infection^7^.

It is followed by a treatise on malaria vaccine^8^ *Salmonella* serovars^9^ and an unfortunate encounter with a cultural related illness - ebinyo in Northern Uganda^10^.

Cardiovascular issues then follow: spontaneous reperfusion in acute ST-segment elevation^11^, adherence to hypertension self-management^12^, non-communicable diseases in Nigeria^13^ and ends with a treatise of on factors related to tip malposition of central catheter in the newborn^14^.

We then have practical papers on cancer^15^,^16^,^17^,^18^,^19^ and the gastrointestinal tract^20^.

Endocrinology then takes the stage with papers on Vitamin D and Hashimoto's thyroiditis^21^, diabetes mellitus,^22^ and injuries and vitamin D deficiency^23^; and gastric bypass in type 2 diabetes mellitus^24^.

Challenges remain none the less. Hence tobacco and alcohol use in pregnancy in Namibia comes to the fore^25^ while treatment of cancer of the cervix in Tanzania is described^26^. Now to HIV that refuses to go away as adolescent experts in Uganda share their experiences of sexual behaviours on HIV^27^; as nurses in China describe application of vertical nursing managers in care of women^28^. Interventions in dentistry^29^, cerebral palsy^30^, bone grafting^31^, and birth asphyxia^32^ follow.

Dementia in Kivu province in Congo^33^ improved on local treatment, as psychological care of undergoing endoscopy^34^ follows. A study on effect of omeprazole on carbapenem resistance Gram negative bacilli highlights some topical innovation^35^ is followed by Lesch-Nyhan syndrome in dental work^36^, and use of CT scan for diagnosis of lung adenocarcinoma^37^. Insights into the efficacy of a herb^38^ using HPLC, computer assisted dentistry^39^, molecular biology of Acute Lymphoblastic Leukemia^40^, and the use of laser in cataract extraction and its impact on functions of the eye^41^ complete this treatise.

We wish you a fruitful reading as you enjoy the new season wherever you might be!
